# Supplementary material for: Polygenic risk scores and Parkinson’s disease in South Africa: Moving towards ancestry-informed disease prediction
Source: medRxiv. 2025 Sep 30:2025.09.29.25336747. Preprint. [Version 1] doi: 10.1101/2025.09.29.25336747 (PMC12622108; doi:10.1101/2025.09.29.25336747)
Supplement: Supplement 1 [file media-1.pdf]

Polygenic risk scores and Parkinson's disease in South Africa: Towards ancestry-informed prediction

Kathryn Step, Carene Anne Alene Ndong Sima, Spencer Grant, Emily Waldo, Soraya Bardien, Ignacio F. Mata on behalf of the Global Parkinson's Genetics Program

Table of Contents

Supplementary figures *4*

Supplementary Figure 1: Karyograms showing the inferred local ancestry of three individuals from the South African Parkinson's Disease Study Collection. This highlights the complex admixture of the cohort. AFR, African; EAS, East Asian; EUR, European; MAL, Malaysian; NAMA, Nama; POP, Population; SAS, South Asian 4

Supplementary Figure 2: The following plots are using Nalls et al 2019 as the base dataset. (A) PRS R^2 across clumping thresholds. The variance explained (R^2) by the polygenic risk score at different GWAS p-value thresholds, stratified by linkage disequilibrium clumping parameters (r^2 and kb). Each panel corresponds to a clumping window size (kb), with points and lines indicating R^2 across p-value thresholds. (B) PRS significance versus predictive performance. Relationship between PRS model significance (PRS association p-value; log-scaled) and variance explained (R^2). Colors indicate clumping window sizes (kb) and shapes indicate LD thresholds (r^2), highlighting the trade-off between model fit and predictive power. 5

Supplementary Figure 3: The following plots are using Kim et al 2024 as the base dataset. (A) PRS R^2 across clumping thresholds. The variance explained (R^2) by the polygenic risk score at different GWAS p-value thresholds, stratified by linkage disequilibrium clumping parameters (r^2 and kb). Each panel corresponds to a clumping window size (kb), with points and lines indicating R^2 across p-value thresholds. (B) PRS significance versus predictive performance. Relationship between PRS model significance (PRS association p-value; log-scaled) and variance explained (R^2). Colors indicate clumping window sizes (kb) and shapes indicate LD thresholds (r^2), highlighting the trade-off between model fit and predictive power. 6

Supplementary Figure 4: Density plots showing the polygenic risk score distribution for cases versus controls. (A) Density plot with Nalls et al 2019 as the base dataset and (B) the density plot with the Kim et al 2024. 6

Supplementary Figure 5: Bars plot showing the proportion of phenotypic variance (R^2) explained by the polygenic risk score (PRS) at varying SNP inclusion p-value thresholds. Each bar represents a PRS model fit calculated at a specific threshold. The optimal threshold—defined as the point with the highest R^2—is highlighted, indicating the most predictive model. (A) Results shown are based on the training dataset using summary statistics from Nalls *et al.*, 2019 for the strongest association. (B) Results shown are based on the training dataset using summary statistics from Nalls *et al.*, 2019 for the highest predictive performance. (C) Results shown are based on the validation dataset using summary statistics from Nalls *et al.*, 2019 based on the highest predictive performance thresholds from the training dataset. 7

Supplementary Figure 6: Bars plot showing the proportion of phenotypic variance (R^2) explained by the polygenic risk score (PRS) at varying SNP inclusion p-value thresholds. Each bar represents a PRS model fit calculated at a specific threshold. The optimal threshold—defined as the point with the highest R^2—is highlighted, indicating the most predictive model. (A) Results shown are based on the training dataset using summary statistics from Kim *et al.*, 2019 for the strongest association. (B) Results shown are based on the training dataset using summary statistics from Kim *et al.*, 2024 for the highest predictive performance. (C) Results shown are based on the validation dataset using summary

statistics from Kim *et al*., 2024 based on the highest predictive performance thresholds from the validation dataset. 7

Supplementary Tables....*8*

Supplementary Table 1: Study participants included in the polygenic risk score analysis 8

Supplementary Table 2: Stability of PRS performance estimates across repeated for 20 random splits ..9

Supplementary Table 3: Positive predictive value and negative predictive value of polygenic risk scores for Parkinson's disease at multiple top-percentile thresholds 10

Supplementary Table 4: Variance explained by polygenic risk score models with different covariate adjustments for the validation datasets 11

Global Parkinson’s Genetics Program banner author list....*11*

Supplementary figures

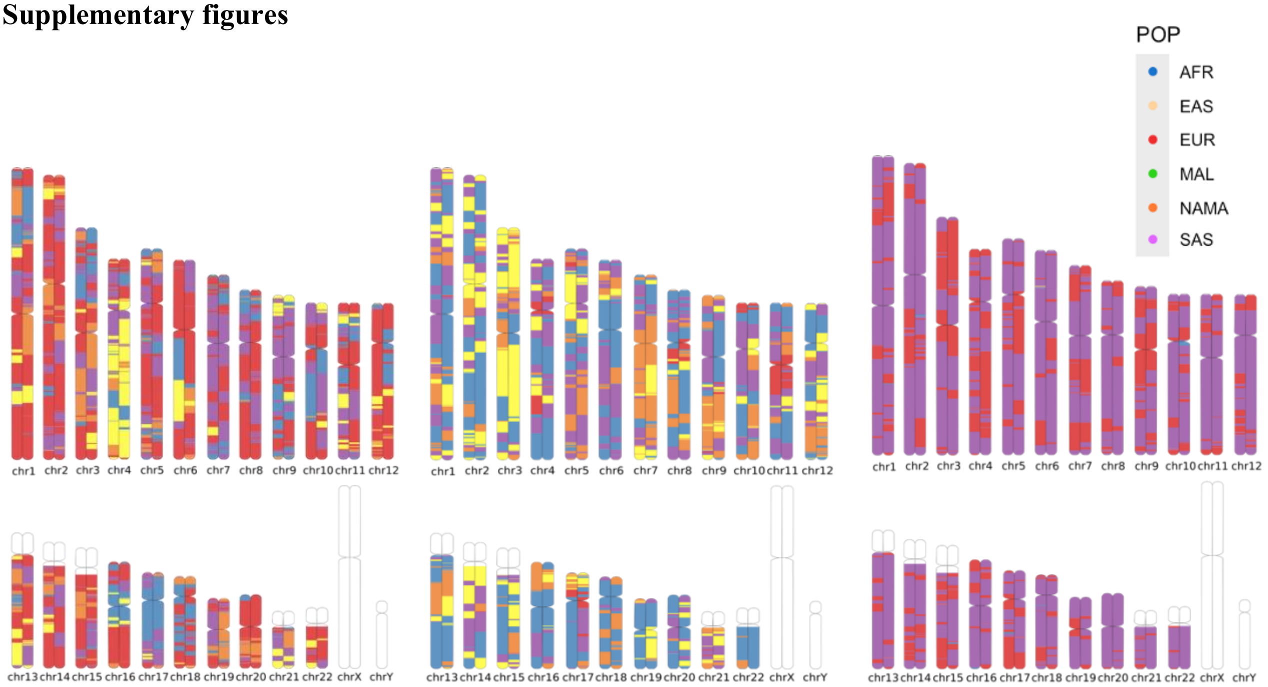

Supplementary Figure 1: Karyograms showing the inferred local ancestry of three individuals from the South African Parkinson's Disease Study Collection. This highlights the complex admixture of the cohort. AFR, African; EAS, East Asian; EUR, European; MAL, Malaysian; NAMA, Nama; POP, Population; SAS, South Asian

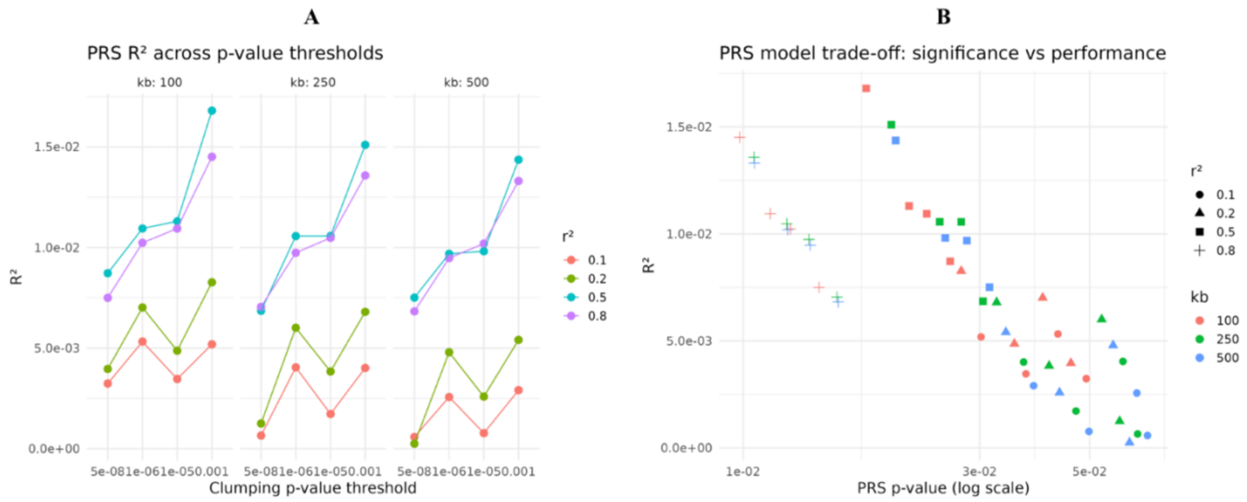

Supplementary Figure 2: The following plots are using Nalls et al 2019 as the base dataset. **(A)** PRS R^2 across clumping thresholds. The variance explained (R^2) by the polygenic risk score at different GWAS p-value thresholds, stratified by linkage disequilibrium clumping parameters (r^2 and kb). Each panel corresponds to a clumping window size (kb), with points and lines indicating R^2 across p-value thresholds. **(B)** PRS significance versus predictive performance. Relationship between PRS model significance (PRS association p-value; log-scaled) and variance explained (R^2). Colors indicate clumping window sizes (kb) and shapes indicate LD thresholds (r^2), highlighting the trade-off between model fit and predictive power.

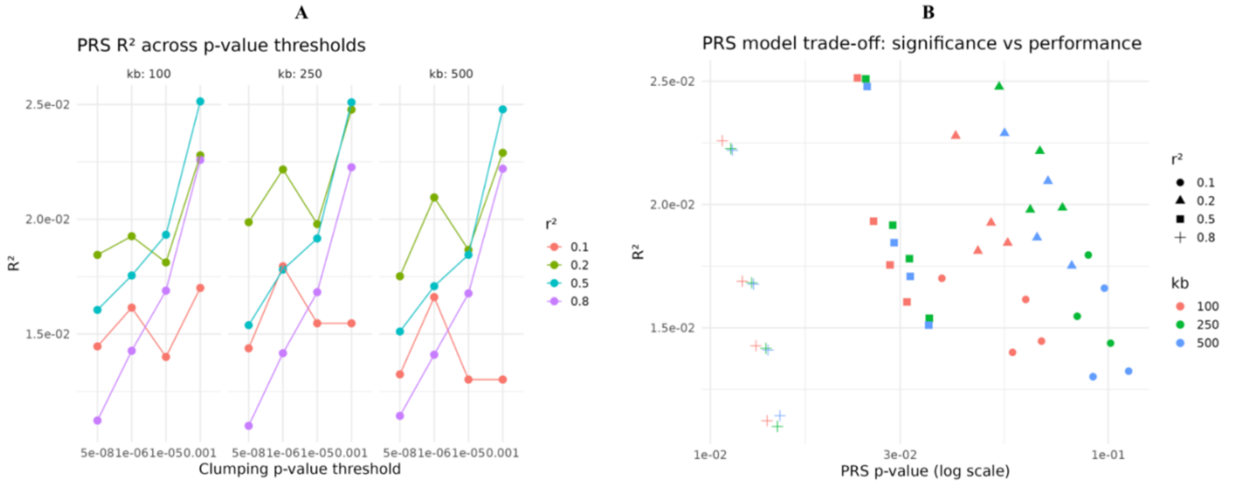

Supplementary Figure 3: The following plots are using Kim et al 2024 as the base dataset. **(A)** PRS R^2 across clumping thresholds. The variance explained (R^2) by the polygenic risk score at different GWAS p-value thresholds, stratified by linkage disequilibrium clumping parameters (r^2 and kb). Each panel corresponds to a clumping window size (kb), with points and lines indicating R^2 across p-value thresholds. **(B)** PRS significance versus predictive performance**.** Relationship between PRS model significance (PRS association p-value; log-scaled) and variance explained (R^2). Colors indicate clumping window sizes (kb) and shapes indicate LD thresholds (r^2), highlighting the trade-off between model fit and predictive power.

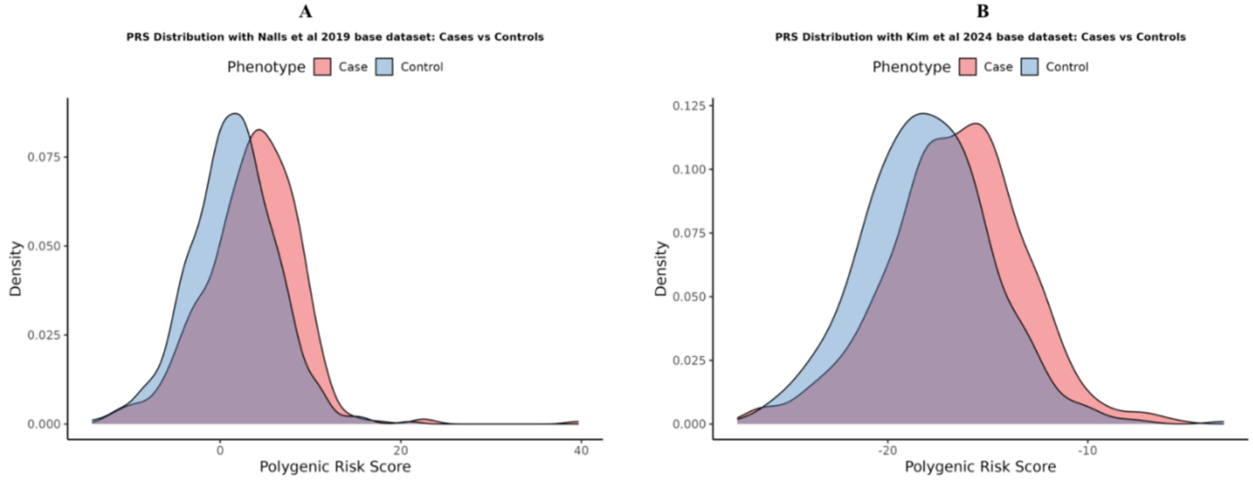

Supplementary Figure 4: Density plots showing the polygenic risk score distribution for cases versus controls. (A) Density plot with Nalls et al 2019 as the base dataset and (B) the density plot with the Kim et al 2024.

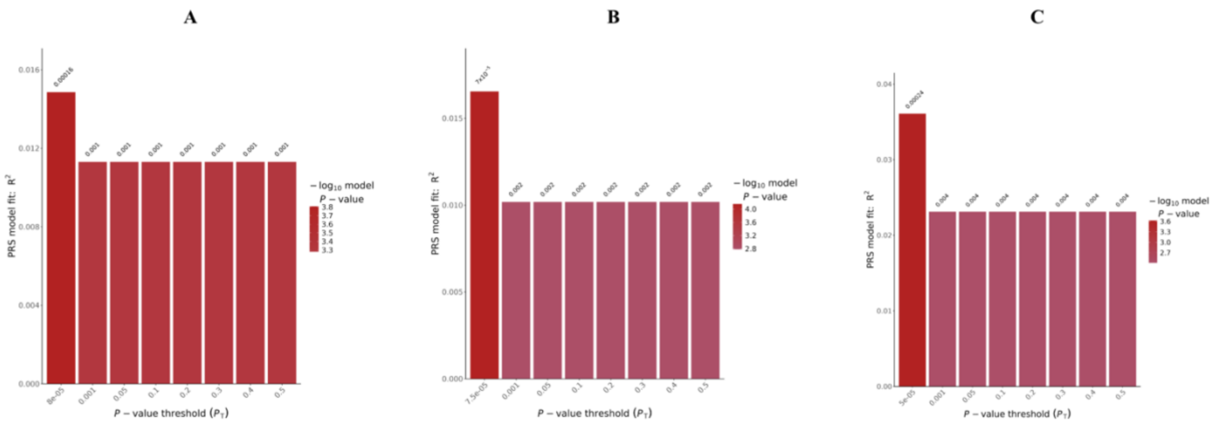

Supplementary Figure 5: Bars plot showing the proportion of phenotypic variance (R^2) explained by the polygenic risk score (PRS) at varying SNP inclusion p-value thresholds. Each bar represents a PRS model fit calculated at a specific threshold. The optimal threshold—defined as the point with the highest R^2—is highlighted, indicating the most predictive model. **(A)** Results shown are based on the training dataset using summary statistics from Nalls *et al*., 2019 for the strongest association. **(B)** Results shown are based on the training dataset using summary statistics from Nalls *et al*., 2019 for the highest predictive performance. **(C)** Results shown are based on the validation dataset using summary statistics from Nalls *et al*., 2019 based on the highest predictive performance thresholds from the training dataset.

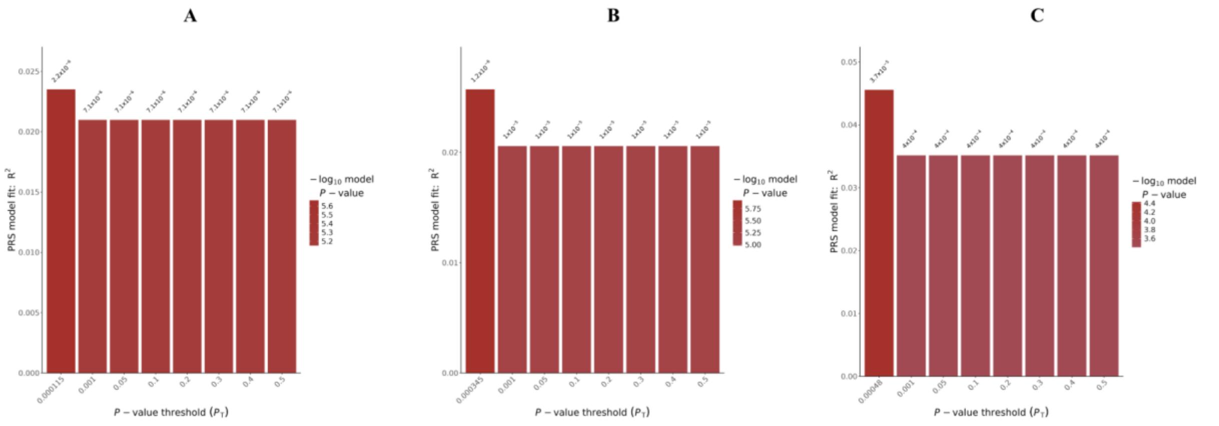

Supplementary Figure 6: Bars plot showing the proportion of phenotypic variance (R^2) explained by the polygenic risk score (PRS) at varying SNP inclusion p-value thresholds. Each bar represents a PRS model fit calculated at a specific threshold. The optimal threshold—defined as the point with the highest R^2—is highlighted, indicating the most predictive model. **(A)** Results shown are based on the training dataset using summary statistics from Kim *et al*., 2019 for the strongest association. **(B)** Results shown are based on the training dataset using summary statistics from Kim *et al*., 2024 for the highest predictive performance. **(C)** Results shown are based on the validation dataset using summary statistics from Kim *et al*., 2024 based on the highest predictive performance thresholds from the validation dataset.

Supplementary Tables

Supplementary Table 1: Study participants included in the polygenic risk score analysis

	**Total participants**	**Number of males**	**Number of females**	**Mean (SD) age**	**Mean (SD) AAO**
**PD cases**	691	398	293	65.77 (12.06)	57.44 (12.56)
**Controls**	826	385	441	44.72 (21.49)	N/A
**Total**	1517	783	734	54.31 (20.67)	N/A
**PD cases retained for analysis**	661	382	279	65.51 (12.08)	57.58 (12.50)
**Controls retained for analysis**	737	346	391	45.43 (21.79)	N/A
**Total retained for analysis**	1398	728	670	54.92 (20.48)	N/A

Legend: The first three rows show the study participant information prior to quality control and removal of related individuals. AAO, age at onset; N/A, not applicable; SD, Standard deviation

Supplementary Table 2: Stability of PRS performance estimates across repeated for 20 random splits

	Nalls et al 2019						Kim et al 2024					
Split	Training AUC	Training AUC CI Lower	Training AUC CI Upper	Validation AUC	Validation AUC CI Lower	Validation AUC CI Upper	Training AUC	Training AUC CI Lower	Training AUC CI Upper	Validation AUC	Validation AUC CI Lower	Validation AUC CI Upper
1	0.6565	0.6225	0.6906	0.5661	0.5100	0.6222	0.6400	0.6054	0.6746	0.5983	0.5436	0.6529
2	0.6279	0.5929	0.6629	0.6243	0.5706	0.6780	0.6294	0.5944	0.6644	0.6237	0.5702	0.6772
3	0.6425	0.6078	0.6772	0.6050	0.5510	0.6590	0.6241	0.5889	0.6592	0.6221	0.5687	0.6755
4	0.6188	0.5836	0.6540	0.6516	0.5991	0.7040	0.6276	0.5927	0.6626	0.6357	0.5823	0.6891
5	0.6361	0.6015	0.6707	0.6261	0.5721	0.6801	0.6353	0.6005	0.6700	0.6248	0.5709	0.6787
6	0.6350	0.6002	0.6698	0.6195	0.5659	0.6732	0.6352	0.6004	0.6700	0.6113	0.5573	0.6653
7	0.6316	0.5967	0.6665	0.6248	0.5713	0.6783	0.6420	0.6075	0.6765	0.6016	0.5465	0.6567
8	0.6188	0.5837	0.6539	0.6573	0.6051	0.7095	0.6200	0.5849	0.6551	0.6429	0.5898	0.6961
9	0.6009	0.5652	0.6365	0.6665	0.6150	0.7180	0.6028	0.5671	0.6385	0.6844	0.6330	0.7357
10	0.6424	0.6078	0.6769	0.5640	0.5092	0.6189	0.6442	0.6095	0.6789	0.6000	0.5458	0.6541
11	0.6172	0.5818	0.6525	0.6609	0.6090	0.7128	0.6082	0.5727	0.6437	0.6694	0.6180	0.7208
12	0.6333	0.5984	0.6682	0.6248	0.5711	0.6784	0.6474	0.6127	0.6820	0.5870	0.5326	0.6414
13	0.6456	0.6111	0.6802	0.6007	0.5465	0.6550	0.6324	0.5973	0.6675	0.6117	0.5579	0.6655
14	0.6363	0.6014	0.6712	0.6094	0.5556	0.6632	0.6342	0.5993	0.6692	0.6124	0.5584	0.6663
15	0.6211	0.5861	0.6562	0.6476	0.5950	0.7002	0.6089	0.5736	0.6442	0.6638	0.6116	0.7160
16	0.6169	0.5816	0.6523	0.6624	0.6108	0.7139	0.6250	0.5899	0.6601	0.6269	0.5735	0.6802
17	0.6354	0.6005	0.6703	0.6124	0.5586	0.6661	0.6346	0.5998	0.6694	0.6179	0.5639	0.6718
18	0.6260	0.5910	0.6610	0.6488	0.5961	0.7014	0.6273	0.5924	0.6623	0.6312	0.5777	0.6847
19	0.6189	0.5836	0.6542	0.6502	0.5978	0.7026	0.6218	0.5867	0.6570	0.6410	0.5880	0.6940
20	0.6110	0.5757	0.6463	0.6735	0.6222	0.7248	0.6163	0.5811	0.6515	0.6478	0.5952	0.7003

Mean ± SD	0.6286 ± 0.0133	0.5936 ± 0.0137	0.6636 ± 0.0130	0.6298 ± 0.0311	0.5766 ± 0.0323	0.6830 ± 0.0299	0.6278 ± 0.1223	0.5928 ± 0.0125	0.6628 ± 0.0119	0.6277 ± 0.0252	0.5743 ± 0.0261	0.6811 ± 0.0242
Legend: AUC, Area Under the Curve; CI, Confidence interval; OR, Odds ratio; R2, Nagelkerke's R^2; SD, Standard deviation												

Supplementary Table 3: Positive predictive value and negative predictive value of polygenic risk scores for Parkinson's disease at multiple top-percentile thresholds

**Threshold**	**Cutoff value**	**Number of cases above threshold**	**Number of controls below threshold**	**Sensitivity**	**Specificity**	**Positive predictive value**	**Negative predictive value**
**Nalls et al 2019**							
95%	9.776	29	514	0.0652	0.9625	0.0024	0.9987
90%	8.337	67	503	0.1506	0.9419	0.0036	0.9987
80%	6.446	125	463	0.2809	0.8670	0.0029	0.9989
**Kim et al 2024**							
95%	-12.091	34	519	0.0764	0.9719	0.0038	0.9987
90%	-13.129	67	503	0.1506	0.9419	0.0036	0.9987
80%	-14.751	125	463	0.2809	0.8670	0.0029	0.9989
Legend: Positive predictive value (PPV), negative predictive value, sensitivity, and specificity were calculated for top 5%, 10%, and 20% of polygenic risk score (PRS) distributions for two base summary statistics: Nalls et al. 2019 and Kim et al. 2024. Sensitivity represents the proportion of cases above the PRS threshold, and specificity represents the proportion of controls below the threshold. PPV and NPV were adjusted for the global Parkinson's disease prevalence of 0.001386. Cutoff values correspond to the PRS score at each percentile threshold.							

Supplementary Table 4: Variance explained by polygenic risk score models with different covariate adjustments for the validation datasets

Study	PRS R2	PRS R2 adjusted	Full R2	Null R2	Number of SNPs	Empirical p-value	Covariates included
Nalls	0.037	0.038	0.356	0.318	486	3.00E-04	Age, Sex, Ancestries
Nalls	0.057	0.055	0.313	0.257	473	1.00E-04	Age
Nalls	0.063	0.052	0.084	0.032	1590	1.00E-04	Sex
Nalls	0.046	0.038	0.082	0.044	1590	5.00E-04	Ancestries
Nalls	0.050	0.049	0.329	0.279	486	1.00E-04	Age, Sex
Nalls	0.040	0.040	0.341	0.301	534	1.00E-04	Age, Ancestries
Nalls	0.042	0.035	0.104	0.069	1590	1.20E-03	Sex, Ancestries
Kim	0.045	0.045	0.363	0.318	969	1.00E-04	Age, Sex, Ancestries
Kim	0.058	0.056	0.314	0.257	990	1.00E-04	Age
Kim	0.059	0.049	0.080	0.032	1831	1.00E-04	Sex
Kim	0.052	0.043	0.086	0.044	1831	2.00E-04	Ancestries
Kim	0.053	0.053	0.332	0.279	969	1.00E-04	Age, Sex
Kim	0.048	0.048	0.349	0.301	969	1.00E-04	Age, Ancestries
Kim	0.049	0.041	0.110	0.069	1831	2.00E-04	Sex, Ancestries

Legend: Table shows PRS R^2 values, adjusted and unadjusted, with corresponding full and null model R^2, empirical significance, and number of SNPs included, stratified by covariates used in each model. PRS R2 is the variance explained by the PRS. PRS R2 adjusted is the PRS R2 adjusted for ascertainment. The Full R2 is the variance explained by the full model (including the covariates). Null R2 is the variance explained by the covariates only,

Global Parkinson's Genetics Program banner author list

Country	Name	Institution	Funders and Disclosures
**Algeria**	Yasser Mecheri	Centre Hospitalo-Universitaire Dr Benbadis Constantine	Nothing to declare
**Argentina**	Emilia M Gatto	Sanatorio de la Trinidad Mitre- INEBA	Nothing to declare
	Marcelo Kauffman	Hospital JM Ramos Mejia	Nothing to declare
	Federico Capparelli	Centro de Educación Médica e Investigaciones Clínicas Norberto Quirno	Nothing to declare

**Armenia**	Samson Khachatryan	Somnus Neurology Clinic	Nothing to declare
	Zaruhi Tavadyan	Somnus Neurology Clinic	Nothing to declare
	Mariam Isayan	Somnus Neurology Clinic	Nothing to declare
**Australia**	Claire E Shepherd	Neuroscience Research Australia	The Sydney Brain Bank is located at and supported by Neuroscience Research Australia
	Kishore Kumar	Garvan Institute of Medical Research and Concord Repatriation General Hospital	Paul Ainsworth Family Foundation
	Melina Ellis	Concord Hospital	Nothing to declare
	Miguel E. Rentería	QIMR Berghofer Medical Research Institute	The Australian Parkinson's Genetics Study is supported by the Shake It Up Australia Foundation and The Michael J. Fox Foundation for Parkinson's Research
	Sulev Koks	Murdoch University	Nothing to declare
	Simon Rowe	Neuroscience Research Australia	Nothing to declare
	Laura Rudaks	Neuroscience Research Australia	Nothing to declare
	Dennis Yeow	Neuroscience Research Australia	Nothing to declare
	Carolyn Sue	Neuroscience Research Australia	Nothing to declare
	Victor Flores Ocampo	QIMR Berghofer Medical Research Institute	Nothing to declare
**Austria**	Alexander Zimprich	Medical University Vienna Austria	Nothing to declare
**Azerbaijan**	Kanan Jafarov	Istanbul Klinik	Nothing to declare
**Belgium**	David Crosiers	University of Antwerp	Nothing to declare

**Brazil**	Artur F. Schumacher-Schuh	Universidade Federal do Rio Grande do Sul / Hospital de Clínicas de Porto Alegre	Nothing to declare
	Carlos Rieder	Federal University of Health Sciences of Porto Alegre	Nothing to declare
	Paula Saffie Awad	Universidade Federal do Rio Grande do Sul	Global Parkinson's Genetics Program
	Vitor Tumas	University of São Paulo	Nothing to declare
	Sarah Camargos	Universidade Federal de Minas Gerais	Nothing to declare
	Lucas Faria Costa	Universidade Federal de Minas Gerais	Nothing to declare
**Canada**	Edward A. Fon	Montreal Neurological Institute	Nothing to declare
	Oury Monchi	Institut universitaire de gériatrie de Montréal	CIHR, Brain Canada, Parkinson Canada
	Edward Fon	McGill University	Nothing to declare
	Robert Thibault	Aligning Science Across Parkinson's	Nothing to declare
	Ziv Gan-Or	McGill University	Nothing to declare
	Anthony Lang	University of Toronto	Nothing to declare
	Konstantin Senkevich	McGill University	Nothing to declare
**Chile**	Benjamin Pizarro Galleguillos	Universidad de Chile	Nothing to declare
	Marcelo Miranda	Fundación Diagnosis	Nothing to declare
	Maria Leonor Bustamante	Faculty of Medicine Universidad de Chile	Nothing to declare
	Patricio Olguin	Universidad de Chile	Nothing to declare
	Pedro Chana	CETRAM	Nothing to declare
	Elias Fernandez	Hospital Regional de Concepción	Nothing to declare
**China**	Beisha Tang	Central South University	Nothing to declare

	Huifang Shang	West China Hospital Sichuan University	Nothing to declare
	Jifeng Guo	Xiangya Hospital	Nothing to declare
	Piu Chan	Capital Medical University	Nothing to declare
	Wei Luo	Zhejiang University	Nothing to declare
	Zhenhua Liu	Xiangya Hospital, Central South University	Nothing to declare
**Colombia**	Gonzalo Arboleda	Universidad Nacional de Colombia	Nothing to declare
	Jorge Orozco	Fundación Valle del Lili	Nothing to declare
	Marlene Jimenez del Rio	University of Antioquia	Nothing to declare
**Costa Rica**	Alvaro Hernandez	University of Costa Rica	Nothing to declare
**Denmark**	Per Borghammer	Aarhus University	Nothing to declare
**Egypt**	Mohamed Salama	The American University in Cairo	The AUC/ ASRT/ DAAD
	Walaa A. Kamel	Beni-Suef University	Nothing to declare
**Ethiopia**	Yared Z. Zewde	Addis Ababa University	Nothing to declare
**France**	Alexis Brice	Paris Brain Institute	Nothing to declare
	Jean-Christophe Corvol	Sorbonne Université	Nothing to declare
	Mari Vidailhet	Salpêtriere Hospital (AP-HP), Sorbonne Université	Nothing to declare
**Georgia**	Mariam Kekenadze	Tbilisi State Medical University	Nothing to declare
	Irine Khatiashvili	S. Khechinashvili University Hospital	Nothing to declare
**Germany**	Ana Westenberger	University of Lübeck	Nothing to declare
	Anastasia Illarionova	Deutsches Zentrum für Neurodegenerative Erkrankungen	Nothing to declare
	Brit Mollenhauer	University Medical Center Göttingen	Nothing to declare

	Christine Klein	University of Lübeck	CK serves as a medical Advisor to Centogene on genetic testing reports in the field of movement disorders, except Parkinson's disease, and is a member of the Scientific Advisory Board of Retromer Therapeutics
	Eva-Juliane Vollstedt	University of Lübeck	Nothing to declare
	Franziska Hopfner	Department of Neurology, University Hospital, LMU Munich	Nothing to declare
	Günter Höglinger	Department of Neurology, University Hospital, LMU Munich	Nothing to declare
	Harutyun Madoev	University of Lübeck	Nothing to declare
	Joanne Trinh	University of Lübeck	Nothing to declare
	Katja Lohmann	University of Lübeck	Nothing to declare
	Lara M. Lange	University of Lübeck and University Medical Center Schleswig-Holstein	Nothing to declare
	Manu Sharma	University of Tubingen	Dr. Sharma is further funded by the Michael J Fox Foundation, USA Genetic Diversity in PD Program: GAP-India Grant ID: 009411.
	Sergiu Groppa	University of Mainz	Nothing to declare
	Thomas Gasser	University of Tübingen	Nothing to declare
	Zih-Hua Fang	The German Center for Neurodegenerative Diseases	Nothing to declare
	Karl Heilbron	Charité - Universitätsmedizin Berlin	Former employee of 23andMe, Inc. Owns stock and/or stock options in 23andMe, Inc.
	Theresa Luth	University of Luebeck	Nothing to declare
	Wenhua Sun	University of Teubingen	Nothing to declare

	Inke König	University of Lübeck	Nothing to declare
	Daniela Berg	University Medical Center Schleswig-Holstein	Nothing to declare
	Bernhard Haslinger	Technical University of Munich	Nothing to declare
	Teresa Kleinz	University of Lübeck	Nothing to declare
	Norbert Brüggemann	University of Lübeck	Dr. Brüggemann received honaria from Abbvie, Esteve, Ipsen, Merz, Teva and Zambon
**Ghana**	Albert Akpalu	University of Ghana Medical School	Nothing to declare
**Greece**	Georgia Xiromerisiou	University of Thessaly	Nothing to declare
	Georgios Hadjigorgiou	University of Thessaly	Nothing to declare
	Ioannis Dagklis	Aristotle University of Thessaloniki	Nothing to declare
	Ioannis Tarnanas	Ionian University	Nothing to declare
	Leonidas Stefanis	Biomedical research Foundation of the Academy of Athens	PPMI2 (funded by MJFF), ALAMEDA (H2020 grant), funding by HFRI
	Maria Stamelou	Diagnostic and Therapeutic Centre HYGEIA Hospital	The non-profit organisation for scientific research in Parkinson's disease and related disorders
	Efthymios Dadiotis	University of Thessaly	Nothing to declare
**Honduras**	Alex Medina	Hospital San Felipe	Nothing to declare
**Hong Kong**	Germaine Hiu-Fai Chan	Queen Elizabeth Hospital	Nothing to declare
	Nancy Ip	The Hong Kong University of Science and Technology	Nothing to declare
	Nelson Yuk-Fai Cheung	Queen Elizabeth Hospital	Nothing to declare

	Phillip Chan	The Hong Kong University of Science and Technology	Nothing to declare
	Xiaopu Zhou	The Hong Kong University of Science and Technology	Nothing to declare
**India**	Asha Kishore	Aster Medcity	Michael J. Fox Foundation
	Divya KP	Sree Chitra Tirunal Institute for Medical Sciences and Technology	Nothing to declare
	Pramod Pal	National Institute of Mental Health & Neurosciences	Nothing to declare
	Prashanth Lingappa Kukkle	Manipal Hospital	Nothing to declare
	Roopa Rajan	All India Institute of Medical Sciences	Nothing to declare
	Rupam Borgohain	Nizam's Institute Of Medical Sciences	Nothing to declare
**Iran**	Mehri Salari	Shahid Beheshti University of Medical Science	Nothing to declare
**Israel**	Tamara Shiner	Tel Aviv Sourasky Medical Center	Nothing to declare
	Avner Thaler	Tel Aviv Sourasky Medical Center	Nothing to declare
**Italy**	Andrea Quattrone	Magna Græcia University of Catanzaro	Nothing to declare
	Enza Maria Valente	University of Pavia	Nothing to declare
	Grazia Annesi	National Research Council	Nothing to declare
	Lucilla Parnetti	University of Perugia	Nothing to declare
	Micol Avenali	University of Pavia	Nothing to declare
	Monica Gagliardi	Magna Graecia University	Nothing to declare
	Tommaso Schirinzi	University of Rome Tor Vergata	Nothing to declare
	Caterina Galandra	IRCCS Mondino Foundation	Nothing to declare
	Anna De Rosa	University of Naples Federico II	Nothing to declare
**Japan**	Manabu Funayama	Juntendo University	Nothing to declare

	Nobutaka Hattori	Juntendo University faculty of medicine	Nothing to declare
	Tomotaka Shiraishi	Jikei University School of Medicine	Nothing to declare
**Kazakhstan**	Altynay Karimova	Institute of Neurology and Neurorehabilitation	Nothing to declare
	Gulnaz Kaishibayeva	Institute of Neurology and Neurorehabilitation	Nothing to declare
	Aigerim Utegenova	West Kazakhstan Marat Ospanov State Medical University	Nothing to declare
	Aigul Yemagambetova	West Kazakhstan Marat Ospanov State Medical University	Nothing to declare
	Vadim Akhmetzhanov	Medline medical center	Nothing to declare
	Seitzhan Aidarov	National Center for Neurosurgery	Nothing to declare
	Tautanova Raushan	Astana Medical University	Nothing to declare
	Bagyzhan Syzdykova Syzdykova	City multi-field hospital No. 1	Nothing to declare
	Dinara Alzhanova	Astana Medical University	Nothing to declare
	Zhanybek Myrzayev	International University of Postgraduate Education	Nothing to declare
	Saltanat Abdraimova	South Kazakhstan Medical Academy	Nothing to declare
**Kyrgyzstan**	Cholpon Shambetova	Kyrgyz State Medical Academy	Nothing to declare
**Luxembourg**	Rejko Krüger	University of Luxembourg	Nothing to declare
	Patrick May	University of Luxembourg	Nothing to declare
**Malaysia**	Ai Huey Tan	University of Malaya	Nothing to declare
	Azlina Ahmad-Annuar	University of Malaya	Nothing to declare

	Mohamed Ibrahim Norlinah	Universiti Kebangsaan Malaysia	Nothing to declare
	Nor Azian Abdul Murad	UKM Medical Molecular Biology Institute	Nothing to declare
	Shahrul Azmin	Universiti Kebangsaan Malaysia Medical Centre	Nothing to declare
	Shen-Yang Lim	University of Malaya	Nothing to declare
	Wael Mohamed	International Islamic University	Nothing to declare
	Yi Wen Tay	University of Malaya	Nothing to declare
	Azalea Tenerife Pajo	University of Malaya	Nothing to declare
**Mexico**	Daniel Martinez-Ramirez	Tecnologico de Monterrey	Nothing to declare
	Mayela Rodriguez-Violante	Instituto Nacional de Neurologia y Neurocirugia	Nothing to declare
	Paula Reyes-Pérez	Universidad Nacional Autónoma de México	Global Parkinson's Genetics Program
	Alejandra Medina Rivera	Universidad Nacional Autónoma de México	Nothing to declare
	Nancy Monroy Jaramillo	Instituto Nacional de Neurología y Neurocirugía	Nothing to declare
**Mongolia**	Bayasgalan Tserensodnom	Mongolian National University of Medical Sciences	Nothing to declare
**Nepal**	Rajeev Ojha	Tribhuvan University	Nothing to declare
**Netherlands**	Wilma Van De Berg	Vanderbilt University Medical Center	Nothing to declare
	Bas Bleom	Radboud University	Nothing to declare
	Bart Van de Warrenburg	Radboud University Medical Center	Nothing to declare
	Lisette Charbonnier	Brain Research and Innovation Center	Nothing to declare

**New Zealand**	Tim J. Anderson	University of Otago	Health Research Council of New Zealand; Ministry of Business Innovation and Employment (New Zealand), Neurological Foundation of New Zealand
	Toni L. Pitcher	University of Otago	Funding - Health Research Council of New Zealand
**Nigeria**	Arinola Sanyaolu	University of Lagos	Nothing to declare
	Njideka Okubadejo	University of Lagos	Michael J Fox Foundation; Tertiary Education Trust Fund (TETFUND) National Research Fund
	Oluwadamilola Ojo	College of Medicine of the University of Lagos	Nothing to declare
	Simon Izuchukwu Ozomma	University of Calabar Teaching Hospital	Nothing to declare
	Kolawole Wahab	University of Ilorin	Nothing to declare
**Norway**	Lasse Pihlstrøm	Oslo University Hospital	Southeastern Regional Health Authority, Norway and Michael J. Fox Foundation
	Manuela Tan	Oslo University Hospital	Southeastern Norway Regional Health Authority Norway and Michael J. Fox Foundation
	Ingeborg Haugesag Lie	Oslo University Hospital	Nothing to declare
	Jodi Maple-Grødem	Stavanger University Hospital	Nothing to declare
**Pakistan**	Shoaib Ur-Rehman	University of Science and Technology Bannu	Nothing to declare
**Peru**	Mario Cornejo-Olivas	Universidad Cientifica del Sur	Michael J. Fox Foundation for Parkinson's Research and Aligning Science

			Across Parkinson's Initiative
**Philippines**	Maria Leila Doquenia	Metropolitan Medical Center	Nothing to declare
	Raymond Rosales	Metropolitan Medical Center	Nothing to declare
**Puerto Rico**	Angel Vinuela	University of Puerto Rico	Nothing to declare
**Russia**	Elena Iakovenko	Research Center of Neurology	Nothing to declare
	Anna Gareeva	Ufa Federal Research Center	Nothing to declare
	Gulnara Akhmadeeva	Ufa Scientific Center	Nothing to declare
	Irina Gilyazova	Russian Academy of Sciences / Bashkir State Medical University	Nothing to declare
**Saudi Arabia**	Bashayer Al Mubarak	King Faisal Specialist Hospital and Research Center	Nothing to declare
	Muhammad Umair	King Abdullah International Medical Research Center	Nothing to declare
**Singapore**	Eng-King Tan	National Neuroscience Institute	Singapore National Medical Research Council (MOH-OFLCG-000207)
	Jia Nee Foo	Nanyang Technological University	Singapore National Medical Research Council (MOH-000559)
	Elaine Chew	Nanyang Technological University	Nothing to declare
**Slovenia**	Vesna van Midden	Ljubljana University Medical Centre	Nothing to declare
**South Africa**	Ferzana Amod	University of KwaZulu-Natal	Nothing to declare
	Jonathan Carr	University of Stellenbosch	Nothing to declare
	Soraya Bardien	Stellenbosch University	National Research Foundation of South Africa[Grant Number 129249]
	Nikita Pillay	University of the Western Cape	Nothing to declare

	Kathryn Step	Stellenbosch University	Nothing to declare
**South Korea**	Beomseok Jeon	Seoul National University Hospital	Nothing to declare
	Yun Joong Kim	Yongin Severance Hospital	Nothing to declare
	Jung Hwan Shin	Seoul National University	Nothing to declare
	Joowon Jang	Seoul National University	Nothing to declare
**Spain**	Esther Cubo	Hospital Universitario Burgos	Nothing to declare
	Ignacio Alvarez	University Hospital Mutua Terrassa	Nothing to declare
	Janet Hoenicka	Institut de Recerca Sant Joan de Deu	Fondo de Investigación Sanitaria, Instituto Salud Carlos III, Grant PI019/00126
	Katrin Beyer	Research Institute Germans Trias i Pujol	Nothing to declare
	Maria Teresa Periñan	Instituto de Biomedicina de Sevilla	Nothing to declare
	Pau Pastor	University Hospital Germans Trias i Pujol	Nothing to declare
	Ruben Fernandez-Santiago	Hospital Clínic de Barcelona	Nothing to declare
	Pilar Gómez Garre	Instituto de Biomedicina de Sevilla	Nothing to declare
	Pablo Mir	Instituto de Biomedicina de Sevilla	Nothing to declare
**Sudan**	Sarah El-Sadig	Faculty of medicine university of Khartoum	Nothing to declare
**Sweden**	Kajsa Brolin	Lund University	Nothing to declare
	Per Svenningsson	Karolinska Institute	Nothing to declare
	Maria Swanberg	Lund University	Nothing to declare
**Switzerland**	Christiane Zweier	Inselspital Bern, University of Bern	Nothing to declare
	Gerd Tinkhauser	University Hospital Bern	Nothing to declare

	Paul Krack	Inselspital Bern, University of Bern	Nothing to declare
**Taiwan**	Chin-Hsien Lin	National Taiwan University Hospital	Nothing to declare
	Hsiu-Chuan Wu	Chang Gung Memorial Hospital	Nothing to declare
	Pin-Jui Kung	National Taiwan University	Global Parkinson's Genetics Program
	Ruey-Meei Wu	National Taiwan University Hospital	Minister of Science and Technology, Taiwan Government; National Taiwan University; Parkinson foundation (USA); Michael J. Fox Foundation
	Yihru Wu	Chang Gung Memorial Hospital	Nothing to declare
**Tajikistan**	Ganieva Manizha	Avicenna Tajik State Medical University	Nothing to declare
**Tunisia**	Rim Amouri	National Institute Mongi Ben Hamida of Neurology	Nothing to declare
	Samia Ben Sassi	Mongi Ben Hmida National Institute of Neurology	Nothing to declare
**Turkey**	A. Nazlı Başak	Koç University	Kirac Foundation and Koc Univ.
	Gencer Genc	Şişli Etfal Training and Research Hospital	Nothing to declare
	Özgür Öztop Çakmak	Koç University	Nothing to declare
	Sibel Ertan	Koç University	Nothing to declare
**United Kingdom**	Alastair Noyce	Queen Mary University of London	Prof. Noyce reports grants from Parkinson's UK, Barts Charity, Cure Parkinson’s, NIHR, Innovate UK, Virginia Keiley benefaction, Alchemab, Aligning Science Across Parkinson’s and Michael J

			Fox Foundation. Consultancy and personal fees from Astra Zeneca, AbbVie, Profile, Roche, Biogen, UCB, Bial, Charco Neurotech, uMedeor and Britannia.
	Alejandro Martínez-Carrasco	University College London	Global Parkinson's Genetics Program
	Anette Schrag	University College London	NIHR UCL/H Biomedical Research Centre
	Anthony Schapira	University College London	ASAP
	Camille Carroll	University of Plymouth	C Carroll receives salary from University of Plymouth, University Hospitals Plymouth NHS Trust and National Institute of Health Research; she has received advisory, consulting, and/or lecture fees from AbbVie, Bial, Lundbeck, Global Kinetics, Britannia and Medscape, and research funding from Parkinson's UK, Edmond J Safra Foundation, National Institute of Health Research and Cure Parkinson’s
	Donald Grosset	University of Glasgow	Tracking Parkinson's (J-1101) is funded by Parkinson's UK
	Eleanor J. Stafford	University College London	Nothing to declare
	Henry Houlden	University College London	Nothing to declare

	Huw R Morris	University College London	Dr Morris is employed by UCL. In the last 12 months he reports paid consultancy from Roche and Amylyx ; lecture fees/honoraria - BMJ, Kyowa Kirin, Movement Disorders Society. Research Grants from Parkinson’s UK, Cure Parkinson’s Trust, PSP Association, CBD Solutions, Drake Foundation, Medical Research Council, Michael J Fox Foundation. Dr Morris is a co-applicant on a patent application related to C9ORF72 - Method for diagnosing a neurodegenerative disease (PCT/GB2012/052140)
	John Hardy	University College London	Nothing to declare
	Kin Ying Mok	Univeristy College London	Nothing to declare
	Mie Rizig	University College London	Nothing to declare
	Nicholas Wood	University College London	ASAP- CRN
	Nigel Williams	Cardiff University	Parkinson's UK
	Olaitan Okunoye	University College London	Nothing to declare
	Rauan Kaiyrzhanov	University College London	Nothing to declare
	Rimona Weil	University College London	Nothing to declare
	Seth Love	University of Bristol	Medical Research Council (UK) MR/T018569/1
	Simona Jasaityte	University College London	Nothing to declare

	Sumit Dey	Queen Mary University of London	The Michael J. Fox Foundation for Parkinson's Research
	Vida Obese	University College London	Nothing to declare
	Spencer Finch	Queen Mary University of London	Nothing to declare
	Valentina Escott-Price	Cardiff University	Nothing to declare
	Hamin Lee	St George's, University of London	Nothing to declare
	Roger Barker	University of Cambridge	Nothing to declare
	Mina Ryten	University College London	Nothing to declare
	Michele Hu	University of Oxford	Nothing to declare
	Laura Parkkinen	University of Oxford	Nothing to declare
	Kailash Bhatia	University College London	Nothing to declare
	Richard Walker	Northumbria Healthcare at NHS Foundation Trust	Nothing to declare
	Steve Gentleman	Imperial College London	Nothing to declare
	Thomas Warner	University College London	Nothing to declare
	David Burn	Newcastle University	Nothing to declare
	Christian Lambert	Imperial College London	Nothing to declare
	Caroline Williams-Gray	University of Cambridge	Nothing to declare
	Chris Morris	Newcastle University	Newcastle University, Alzheimer's Society, Alzheimer's Research UK, Medical Research Council
	Deborah Attuah	YLD	Nothing to declare
	Raquel Real	University College London	Nothing to declare

	Yen Tai	Imperial College London	Nothing to declare
USA	Alberto Espay	University of Cincinnati	Nothing to declare
	Alyssa O'Grady	The Michael J. Fox Foundation for Parkinson's Research	Nothing to declare
	Andrew B Singleton	National Institute on Aging	Michael J Fox Foundation for Parkinson's disease Research and Aligning Science Across Parkinson's Initiative
	Andrew K. Sobering	Augusta University / University of Georgia Medical Partnership	Nothing to declare
	Bernadette Siddiqi	The Michael J. Fox Foundation for Parkinson's Research	Nothing to declare
	Bradford Casey	The Michael J. Fox Foundation for Parkinson's Research	Nothing to declare
	Brian Fiske	The Michael J. Fox Foundation for Parkinson's Research	Nothing to declare
	Cabell Jonas	Mid-Atlantic Permanente Medical Group	Nothing to declare
	Carlos Cruchaga	Washington University	National Institutes of Health (R01AG044546 (CC), P01AG003991(CC, JCM), RF1AG053303 (CC), RF1AG058501 (CC), U01AG058922 (CC), RF1AG074007 (YJS)), the Chuck Zuckerberg Initiative (CZI), the Michael J. Fox Foundation (LI, CC), and the Department of Defense (LI- W81XWH2010849). The recruitment and clinical characterization of research participants at Washington University were supported by NIH P30AG066444 (JCM),

			P01AG03991(JCM), and P01AG026276(JCM).
	Caroline B. Pantazis	National Institutes of Health	Nothing to declare
	Charisse Comart	The Michael J. Fox Foundation for Parkinson's Research	Nothing to declare
	Claire Wegel	Indiana University	Nothing to declare
	Cornelis Blauwendraat	National Institutes of Health	Nothing to declare
	Dan Vitale	National Institutes of Health	Nothing to declare
	Deborah Hall	Rush University	Nothing to declare
	Dena Hernandez	National Institutes of Health	Nothing to declare
	Ejaz Shiamim	Kaiser Permanente	Nothing to declare
	Ekemini Riley	Coalition for Aligning Science	Nothing to declare
	Faraz Faghri	National Institutes of Health	F.F.'s participation in this research was supported in part by the Intramural Research Program of the NIH, National Institute on Aging (NIA), National Institutes of Health, Department of Health and Human Services; project number ZO1 AG000535, as well as the National Institute of Neurological Disorders and Stroke. F.F.'s participation in this project was part of a competitive contract awarded to Data Tecnica International LLC by the National Institutes of

			Health to support open science research.
	Geidy E. Serrano	Banner Sun Health Research Institute	Banner Sun Health Research Institute Brain and Body Donation Program of Sun City, Arizona for the provision of human biological materials. The Brain and Body Donation Program has been supported by the National Institute of Neurological Disorders and Stroke (U24 NS072026 National Brain and Tissue Resource for Parkinson’s Disease and Related Disorders), the National Institute on Aging (P30 AG19610 and P30AG072980, Arizona Alzheimer’s Disease Center), the Arizona Department of Health Services (contract 211002, Arizona Alzheimer’s Research Center), the Arizona Biomedical Research Commission (contracts 4001, 0011, 05-901 and 1001 to the Arizona Parkinson's Disease Consortium) and the Michael J. Fox Foundation for Parkinson’s Research

	Hampton Leonard	National Institute on Aging/National Institutes of Health	H.L.L is supported by a competitive contract awarded to Data Tecnica International LLC by the National Institutes of Health to support open science research
	Hirotaka Iwaki	Data Tecnica International	H.I is supported by a competitive contract awarded to Data Tecnica International LLC by the National Institutes of Health to support open science research
	Honglei Chen	Michigan State University	NIH/DoD/Parkinson Foundation/MSU Foundation/Gibby vs. Parky Foundation - No COI to disclose
	Ignacio F. Mata	Cleveland Clinic	Funding from MJFF and NIH
	Ignacio Juan Keller Sarmiento	Northwestern University	Nothing to declare
	Jared Williamson	Kaiser Permanente	Nothing to declare
	Jonggeol Jeff Kim	National Institutes of Health	Nothing to declare
	Joseph Jankovic	Baylor College of Medicine	Nothing to declare
	Joshua Shulman	Baylor College of Medicine / Texas Children's Hospital	Collection of samples and data at Baylor College of Medicine was supported by Huffington Foundation.
	Justin C. Solle	The Michael J. Fox Foundation for Parkinson's Research	Nothing to declare
	Kaileigh Murphy	The Michael J. Fox Foundation for Parkinson's Research	Nothing to declare

	Kamalini Ghosh Galvelis	Parkinson's Foundation	Nothing to declare
	Karen Nuytemans	University of Miami Miller School of Medicine	This work has been supported by the American Parkinson Disease Association and the Margaret Q. Landenberger Research Foundation.
	Karl Kieburtz	Beth Israel Deaconess Medical Center	Nothing to declare
	Kate Andersh	National Institute on Aging	Nothing to declare
	Katerina Markopoulou	North Shore University Health System	Nothing to declare
	Kenneth Marek	Institute for Neurodegenerative Disorders	Consultant for Michael J Fox Foundation, GE Healthcare, Roche, UCB, BIAL, Denali, Takeda, , Cerapsir, UCB, Biohaven, Neuron23, Aprinoia, Astellas, Calico, Genentech, Invicro
	Kristin S. Levine	Data Tecnica International	Nothing to declare
	Lana M. Chahine	University of Pittsburgh	Nothing to declare
	Laura Ibanez	Washington University	Nothing to declare
	Laurel Screven	National Institute on Aging	Nothing to declare
	Lauren Ruffrage	University of Alabama at Birmingham	Nothing to declare
	Lisa Shulman	University of Maryland	Nothing to declare
	Luca Marsili	University of Cincinnati	Nothing to declare
	Maggie Kuhl	The Michael J. Fox Foundation for Parkinson's Research	Nothing to declare

	Marissa Dean	University of Alabama at Birmingham	Dr. Dean is an investigator in studies funded by Abbvie, Inc., Hoffmann-La Roche, CHDI Foundation, Inc., Annexon, Inc., Retrophin, Inc, Neurocrine Biosciences, UniQure Biopharma B.V., Praxis Precision Medicines, Neuraly, Inc., Michael J. Fox Foundation for Parkinson's Research, and US Army Medical Research and Material Command (grant#W81XWH-18-1-0508). In addition, Dr. Dean receives support through the Huntington's Disease Society of American Centers of Excellence program.
	Mary B Makarious	National Institutes of Health	Nothing to declare
	Matthew Farrer	University of Florida - Neurology	Nothing to declare
	Mathew Koretsky	National Institutes of Health	Nothing to declare
	Megan J. Puckelwartz	Northwestern University	Nothing to declare
	Miguel Inca-Martinez	Cleveland Clinic	Nothing to declare
	Mike A. Nalls	National Institutes of Health	M.A.N.'s participation in this project was part of a competitive contract awarded to Data Tecnica International LLC by the National Institutes of Health to support open science research. M.A.N. also currently serves as an advisor for Clover

			Therapeutics and Neuron23 Inc.
	Naomi Louie	The Michael J. Fox Foundation for Parkinson's Research	Nothing to declare
	Niccolò Emanuele Mencacci	Northwestern University	Nothing to declare
	Roger Albin	Universit of Michigan	P50NS123067; Parkinson's Foundation
	Roy Alcalay	Columbia University	Dr. Alcalay is funded by the Micharl J. Fox Foundation and the Parkinson's Foundation. He received consultation fees from Avrobio, Caraway, GSK, Merck, Sanofi, Ono Therapeutics and Takeda
	Ruth Walker	James J. Peters Veterans Affairs Medical Center	Research funding from the Department of Veterans Affairs (CSRD Merit Award CX002342), consulted for Teladoc, Inc. and received an honorarium from New York University (NYU) Medical Center.
	Sara Bandres-Ciga	National Institutes of Health	Nothing to declare
	Sohini Chowdhury	The Michael J. Fox Foundation for Parkinson's Research	Nothing to declare
	Sonya Dumanis	Aligning Science Across Parkinson's	Nothing to declare
	Steven Lubbe	Northwestern University	Nothing to declare
	Tao Xie	University of Chicago	Nothing to declare
	Tatiana Foroud	Indiana University School of Medicine	The Michael J. Fox Foundation for Parkinson's Research

	Thomas Beach	Sun Health Research Institution	Banner Sun Health Research Institute Brain and Body Donation Program of Sun City, Arizona for the provision of human biological materials. The Brain and Body Donation Program has been supported by the National Institute of Neurological Disorders and Stroke (U24NS072026 National Brain and Tissue Resource for Parkinson's Disease and Related Disorders), the National Institute on Aging (P30 AG19610 and P30AG072980, Arizona Alzheimer's Disease Center), the Arizona Department of Health Services (contract 211002, Arizona Alzheimer's Research Center), the Arizona Biomedical Research Commission (contracts 4001, 0011, 05-901 and 1001 to the Arizona Parkinson's Disease Consortium) and the Michael J. Fox Foundation for Parkinson's Research
	Todd Sherer	The Michael J Fox Foundation for Parkinson's Research	Nothing to declare
	Yeajin Song	National Institutes of Health	Nothing to declare
	Dana Lewis	Aligning Science Across Parkinson's	Nothing to declare
	Shreya Menon	Gladstone Institutes	Nothing to declare

	Melissa Nirenberg	Icahn School of Medicine at Mount Sinai	Nothing to declare
	Spencer Grant	National Institutes of Health	Nothing to declare
	Shannon Ballard	National Institutes of Health	Nothing to declare
	Chad Shaw	Baylor College of Medicine	Nothing to declare
	Sidra Aslam	Banner Health	Nothing to declare
	Geidy Serrano	Banner Sun Health Research Institute	Nothing to declare
	Devin Sharp	Aligning Science Across Parkinson's	Nothing to declare
	Kensuke Daida	National Institute of Aging	Nothing to declare
	Rachel Saunders-Pullman	Icahn School of Medicine at Mount Sinai	Nothing to declare
	Michiko Kimura Bruno	The Queen's Medical Center	Nothing to declare
	Matt Farrer	University of Florida College of Medicine	Nothing to declare
	Haydeh Payami	The University of Alabama at Birmingham Heersink School of Medicine	Nothing to declare
	Ryan Pfingst	The Michael J Fox Foundation	Nothing to declare
	James B Leverenz	Cleveland Clinic	Nothing to declare
	Elizabeth Disbrow	LSU Health Shreveport	Nothing to declare
	Debi Brooks	The Michael J Fox Foundation	Nothing to declare
	Randy Schekman	University of California, Berkeley	Nothing to declare
	Un Kang	NYU Grossman School of Medicine	Nothing to declare
	Zbigniew K. Wszolek	Mayo Clinic College of Medicine	Nothing to declare

	Cyrus Zabetian	VA Puget Sound Health Care System	Nothing to declare
	Zach Chaney	The Michael J Fox Foundation	Nothing to declare
	Mark Cookson	National Institute of Health	Nothing to declare
	Christine Swanson-Fischer	National Institute of Health	Nothing to declare
	Conor Hennessey	The Michael J Fox Foundation	Nothing to declare
	Cassandra Barrett	The Michael J Fox Foundation	Nothing to declare
	Beate Ritz	University of California, Los Angeles	Nothing to declare
	Bradley Boeve	Mayo Clinic	Nothing to declare
	Ashley Rawls	University of Florida College of Medicine	Nothing to declare
**Vietnam**	Duan Nguyen	Hue University	Nothing to declare
	Toan Nguyen	Hue University	Nothing to declare
**Zambia**	Masharip Atadzhanov	University of Zambia	Nothing to declare
